# Time series analysis of temporal trends in hemorrhagic fever with renal syndrome morbidity rate in China from 2005 to 2019

**DOI:** 10.1038/s41598-020-66758-4

**Published:** 2020-06-15

**Authors:** Yongbin Wang, Chunjie Xu, Weidong Wu, Jingchao Ren, Yuchun Li, Lihui Gui, Sanqiao Yao

**Affiliations:** 1 Department of Epidemiology and Health Statistics, School of Public Health, Xinxiang Medical University, Xinxiang, Henan Province 453003 P.R. China; 2 Department of Occupational and Environmental Health, School of Public Health, Capital Medical University, Beijing, 100069 P.R. China

**Keywords:** Preventive medicine, Infectious diseases

## Abstract

Hemorrhagic fever with renal syndrome (HFRS) is seriously endemic in China with 70%~90% of the notified cases worldwide and showing an epidemic tendency of upturn in recent years. Early detection for its future epidemic trends plays a pivotal role in combating this threat. In this scenario, our study investigates the suitability for application in analyzing and forecasting the epidemic tendencies based on the monthly HFRS morbidity data from 2005 through 2019 using the nonlinear model-based self-exciting threshold autoregressive (SETAR) and logistic smooth transition autoregressive (LSTAR) methods. The experimental results manifested that the SETAR and LSTAR approaches presented smaller values among the performance measures in both two forecasting subsamples, when compared with the most extensively used seasonal autoregressive integrated moving average (SARIMA) method, and the former slightly outperformed the latter. Descriptive statistics showed an epidemic tendency of downturn with average annual percent change (AAPC) of −5.640% in overall HFRS, however, an upward trend with an AAPC = 1.213% was observed since 2016 and according to the forecasts using the SETAR, it would seemingly experience an outbreak of HFRS in China in December 2019. Remarkably, there were dual-peak patterns in HFRS incidence with a strong one occurring in November until January of the following year, additionally, a weak one in May and June annually. Therefore, the SETAR and LSTAR approaches may be a potential useful tool in analyzing the temporal behaviors of HFRS in China.

## Introduction

Hemorrhagic fever with renal syndrome (HFRS) is a rodent-borne contagious disease caused by several distinct families of Hantaviruses, which can lead to various degrees of fever, shock, congestion, bleeding, and acute renal failure^1^. Currently, this disease globally occurs in more than seventy countries, and an approximate 70%~90% notification was reported in China^2^, where HFRS is still considered a serious public health problem due to its highly endemic in 28 of 31 provinces, municipal districts and autonomous regions with about 20,000–50,000 incident cases per year^3–5^, leading to a fatality rate of around 3%~10%^6^, despite many efforts, such as effective rodent control, vaccination, and environmental management, in reducing HFRS-related incidence over the past decades^7^. In China, the pathogenic agents of HFRS predominantly include Hantaan virus (HTNV) and Seoul virus (SEOV), though other viruses can be involved^4,8,9^. Since 1990s, under the intervention-driven strategies, the notified HFRS cases have begun to decline^2^, but its epidemic trends of HFRS seemingly show a recurring sign in recent years^9–13^. Therefore, to facilitate to offer a quantitative and explicit direction for the prevention and control of HFRS, a forecasting model with strong robustness and high accuracy to understand its epidemic trajectories is required.

At present, many forecasting methods that act as effective policy-supportive tools have widely been adopted to assess and analyze the temporal patterns of the incidence of contagious diseases, such as pertussis^14^, HFRS^3^, pulmonary tuberculosis^15^, influenza^16^, syphilis^17^, etc. Of them, the most commonly used model is the seasonal autoregressive integrated moving average (SARIMA) method that essentially belongs to a linear model^3,17^. However, what is most often encountered in practice is that the data-generating process is highly nonlinear, especially for the morbidity series of infectious diseases because such data often include complicated traits of seasonality, secular trend, cyclicity, and stochastic fluctuation^15,18^. At this time, the linear methods simulated to such complicated nonlinear data frequently fail to obtain satisfactory forecasting performance, whereas the nonlinear methods may do better in that they can better capture the underlying dynamic mechanism of the target series^18,19^. Currently, numerous nonlinear techniques have been recommended to evaluate and analyze the temporal patterns of the incidence of contagious diseases, such as artificial neural networks (ANN_S_)^19^, support vector machine (SVM)^20^, autoregressive conditional heteroscedasticity (ARCH)^21^, Error-Trend-Seasonal (ETS) approaches^22^, etc. However, the popular non-linear regime-switching models such as self-exciting threshold autoregressive (SETAR) and logistic smooth transition autoregressive (LSTAR) specifications so far remain unexplored for the incidence time series forecasting of contagious diseases. Therefore, in the setting of the epidemic status of HFRS in China, the aim is to investigate their forecasting abilities of the SETAR and LSTAR approaches to the HFRS incidence data. Meanwhile, their predictive powers were compared with the SARIMA method to detect the best-performing one that can act as an effective policy-supportive tool for the prevention and control of HFRS.

## Materials and Methods

### Data source

**Figure 1:**
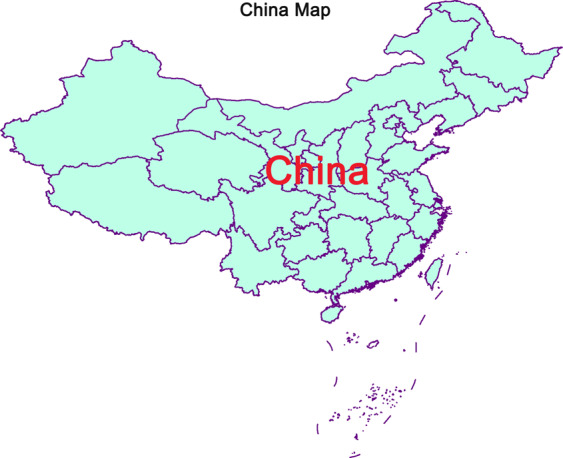
Geographical distribution of China (Created by ArcGIS 10.4.1). Note: The basic geographic information data of China were downloaded from the National Geomatics Center of China (Available at: http://www.ngcc.cn/ngcc/. Accessed on 5 May, 2020).

In this time series analysis, the monthly new cases of HFRS from January 1, 2005 through September 31, 2019 were collected from the national notifiable infectious disease surveillance system (NNIDSS), and the population data were extracted from National Bureau of Statistics (http://www.stats.gov.cn/tjsj/ndsj/). In China (The geographical distribution of China is shown in Fig. 1), the clinically diagnosed or laboratory-confirmed HFRS cases based on the diagnostic criteria for notifiable infectious diseases must be registered on the NNIDSS within 24 hours. A case was confirmed based on the following diagnostic principles^23^: 1) Epidemiological exposure histories. A person has a history of living in the epidemic area or has a direct or indirect contact history with the infected rodents or their excreta (such as feces, saliva, and urine) and secretions within 2 months before the onset of this disease. 2) Clinical manifestations. A person is characterized by gastrointestinal symptoms (such as asthenia, nausea, vomiting, abdominal pain, and diarrhea) and manifestations of capillary damage (such as hyperemia, exudation, and hemorrhage), coupled with hypotension shock or renal damage. 3) Laboratory test. The person with at least one of the laboratory test results in addition to the above 1) and 2) can be confirmed: a positive result for the serum specific IgM antibody, or a 4-time increment for the serum specific IgG antibody in convalescent period than in acute period, or hantavirus RNA detected from the patients, or hantavirus isolated from the patients. We obtained all the data in an anonymous format, without access to any initial information identifying patients, and thus the ethical approval was not needed.

### Building SARIMA model

Owing to the seasonal variation of infectious diseases, the SARIMA approach was often built to simulate and forecast their epidemic levels^5^. This approach is composed of seasonal and non-seasonal parts and can be written as SARIMA(p, d, q)(P, D, Q)_s_, in which p, d, and q signify the non-seasonal autoregressive (AR) order, the non-seasonal differenced times, and the non-seasonal moving average (MA) order, respectively; P, D, and Q represent the seasonal AR (SAR) order, the seasonal differenced times, and the seasonal MA (SMA) order, respectively; S denotes the length of seasonal pattern (S = 12 in this work)^5^. The development of the SARIMA approach was followed by four procedures: Initially, we judged whether the HFRS morbidity series was stationary by plotting its sequence graph and performing an augmented Dickey-Fuller (ADF) test^22,24^. If a nonstationary series was shown, the transformed techniques including logarithm or square root, or/and difference were employed to make it stationary^19^. Secondly, the autocorrelation function (ACF) and partial autocorrelation function (PACF) diagrams were applied to choose its plausible parameters of this model^3^. Subsequently, we determined the preferred SARIMA approach. Among the possible models, the one that presented the lowest values of the Bayesian information criterion (BIC) and Akaike information criterion (AIC), together with the maximum value of the Log-likelihood was considered as the best-fitting^22^. Finally, we further conducted a checking for its parameters and residuals of this optimal model. Once all parameters displayed statistical significances (*p* < 0.05) and the residuals showed a white noise series under the Ljung-Box test (*p* > 0.05), meaning that this best-undertaking SARIMA model can be used to perform forecasting^19^. Otherwise, the above-mentioned modeling steps should be repeated until the best model was found.

### Developing regime-switching models

Due to the data-generating process that is often highly nonlinear, which results in an increasing interest in nonlinear techniques modeled to time series^18^. Of these techniques, the regime-switching methods are significantly popular because they are apt to evaluate and interpret, and capable of producing interesting nonlinearities and rich dynamics^25,26^. These models describe a class of nonlinear regression featuring piecewise linear specifications and regime switching, and are commonly divided into two categories based on the transition function^27^: it is called the SETAR method when using the first-order exponential function; another is called the LSTAR method that uses the logistic function. Both methods have the characteristics of asymmetric cycle^27^. Among them, the LSTAR method allows the expansion and contraction regimes to possess various dynamics, with a smooth transition from one to another. Instead, the SETAR method indicates that different regimes have similar dynamics, whereas the pattern in the transition period may be varied when the process crosses the corresponding threshold^27^. The formula of a two-regime SETAR (2, *p*_1_, *p*_2_) method with delay *d* can be written as^28^.

1 $${Y}_{t}=\{\begin{array}{c}{\phi }_{1,0}+{\phi }_{1,1}{Y}_{t-1}+\cdots +{\phi }_{1,{p}_{1}}{Y}_{t-{p}_{1}}+{\sigma }_{1}{e}_{t}\,if\,{Y}_{t-d}\le r\\ {\phi }_{2,0}+{\phi }_{2,1}{Y}_{t-1}+\cdots +{\phi }_{2,{p}_{2}}{Y}_{t-{p}_{2}}+{\sigma }_{2}{e}_{t}\,if\,{Y}_{t-d} > r\end{array}$$

where *p*_1_ and *p*_2_ represent the autoregressive orders of these two submodels, respectively; *d* denotes the delay parameter; *r* is the threshold value. Further, this representation can be extended to three or more regimes.

The formula of a two-regime LSTAR (2, *p*_1_, *p*_2_) method with delay *d* can be defined as^29^.

2 $$\begin{array}{c}=\,({\phi }_{1,0}+{\phi }_{1,1}{Y}_{t-1}+\cdots +{\phi }_{1,{p}_{1}}{Y}_{t-{p}_{1}})(1-G({Z}_{t},r,th))\\ \,+\,({\phi }_{2,0}+{\phi }_{2,1}{Y}_{t-1}+\cdots +{\phi }_{2,{p}_{2}}{Y}_{t-{p}_{2}})G({Z}_{t},r,th)+\sigma {e}_{t}\end{array}$$

where the *p*_1_, *p*_2_, and *r* have the same meanings described in the SETAR method; $G({Z}_{t},r,th)$ is the logistic function, its location and scale parameters are *th* and 1*/r*, respectively.

In this research, the preferred SETAR model was selected based on the pooled AIC = AIC (low regime model) +AIC (high regime model), a lower value frequently corresponded to the best-fitting model, but a close pooled AIC value was very competitive, which should also be tried. The optimal LSTAR model was chosen on the basis of the AIC and BIC values, in which the one that had lower values of both two indices was the best-undertaking.

### Performance comparison

We used four statistical measures of the mean absolute deviation (MAD), the mean absolute percentage error (MAPE), the root mean squared error (RMSE), and the mean error rate (MER) to evaluate the accuracy of the forecasts among methods. Typically, the method that presented the lowest value among the above-mentioned measures should be deemed as the optimal.

3 $${\rm{MAD}}=\frac{1}{N}\mathop{\sum }\limits_{i=1}^{N}|{Y}_{i}-{\hat{Y}}_{i}|$$

4 $${\rm{MAPE}}=\frac{1}{N}\mathop{\sum }\limits_{i=1}^{N}\frac{|{Y}_{i}-{\hat{Y}}_{i}|}{{Y}_{i}}$$

5 $${\rm{RMSE}}=\sqrt{\frac{1}{N}\mathop{\sum }\limits_{i=1}^{N}{({Y}_{i}-{\hat{Y}}_{i})}^{2}}$$

6 $${\rm{MER}}=\frac{\frac{1}{N}\mathop{\sum }\limits_{i=1}^{N}|{Y}_{i}-{\hat{Y}}_{i}|}{{\bar{Y}}_{i}}$$

where ${Y}_{i}$ stands for the original HFRS incidence values, ${\hat{Y}}_{i}$ denotes the forecasts from the three models, ${\bar{Y}}_{i}$ signifies the mean of the original values, $N$ represents the number of forecasts.

### Statistical process

In this study, we classified the observed series into training and testing subsets, among which the observed series between January 1, 2005 and December 31, 2018 (training subset) was used to fit the models, and then selecting the optimal models to forecast the rest of data (testing subset). Meanwhile, an additional training subset from January 1, 2005 and December 31, 2017 and testing subset from January 1, 2018 to September 31, 2019 were provided to account for the models’ uncertainty. The SARIMA, SETAR, and LSTAR methods were erected using the statistical packages of “forecast,” “fUnitRoots,” “TSA,” “tsDyn” and “tseries” of R3.4.3 (R Development Core Team, Vienna, Austria). Additionally, we detected the nonlinearity of the HRFS morbidity series by applying a Brock-Dechert-Scheinkman (BDS) test to the errors of the optimal SARIMA approach^30^, and using a Lagrangian Multiplier (LM) test to examine whether there existed conditional heteroskedastic behavior and volatility (ARCH effect) in the residual sequence yielded by these three models^22^. A two-sided *p* < 0.05 suggests a statistical significance.

## Results

### Statistical description

**Figure 2:**
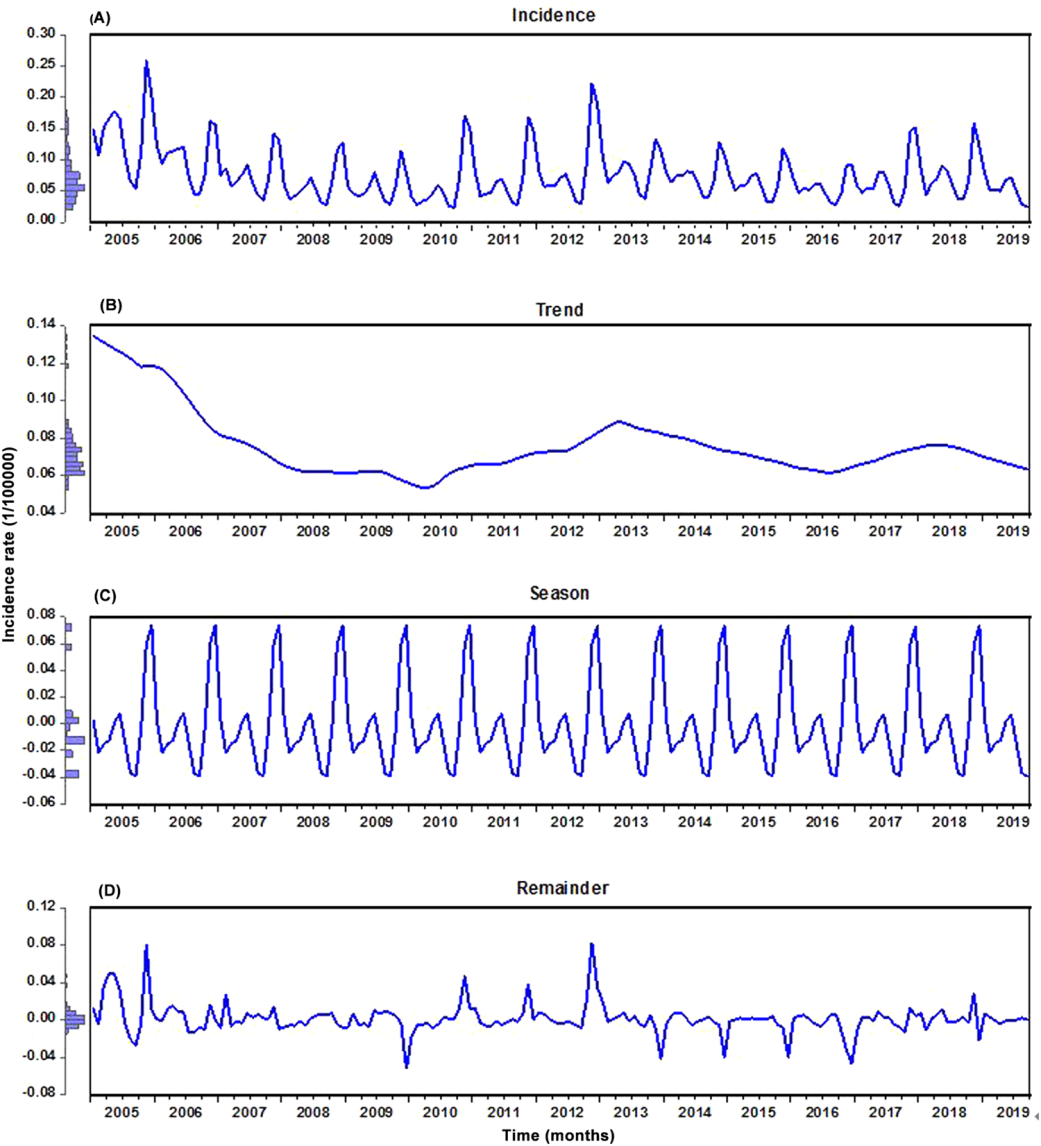
Time series decomposed plots of hemorrhagic fever with renal syndrome (HFRS) morbidity using the STL technique. The HFRS morbidity series was decomposed into three components. (**A**) The actual observed series; (**B**) Trend; (**C**) Seasonal variation; (**D**) Irregular component. As illustrated, there was a pronounced seasonal trait in the HFRS morbidity series.

Throughout the study period, the reported HFRS cases totaled 181,402, resulting in an annualized and monthly morbidity rates of 0.924 and 0.076 per 100,000 persons, respectively. The original incidence series and the decomposition of this series into trend, seasonal pattern, and irregular component are displayed in Fig. 2 **and** Supplementary Fig. S1, indicating that together HFRS incidence displayed a downward trend with average annual percent change (AAPC) of −5.640%, and yet the variation trend seemed to show a natural cyclical pattern with 3–5 years’ fluctuations: morbidity rate dramatically dropped from 1.704 to 0.690 per 100,000 persons in the period 2005–2009, with AAPC = − 19.029%; then it climbed to 1.028 per 100,000 persons in 2012, with AAPC = 9.037% relative to the level of 2009; immediately afterward the trend was decreasing between 2012 and 2016 (1.028 to 0.671 per 100,000 population), with AAPC = − 2.793%; and then with an AAPC = 1.213% from 2016 to 2018. And the HFRS incidence series was strongly seasonal with a cycle of 12 months, where a semi-annual seasonal pattern was observed, with a strong peak occurring from November to January of the following year and a weak one in May and June annually, while a trough was observed in August and September per year (Fig. 2 and Supplementary Fig. S2**)**.

### The best-performing SARIMA method

**Figure 3:**
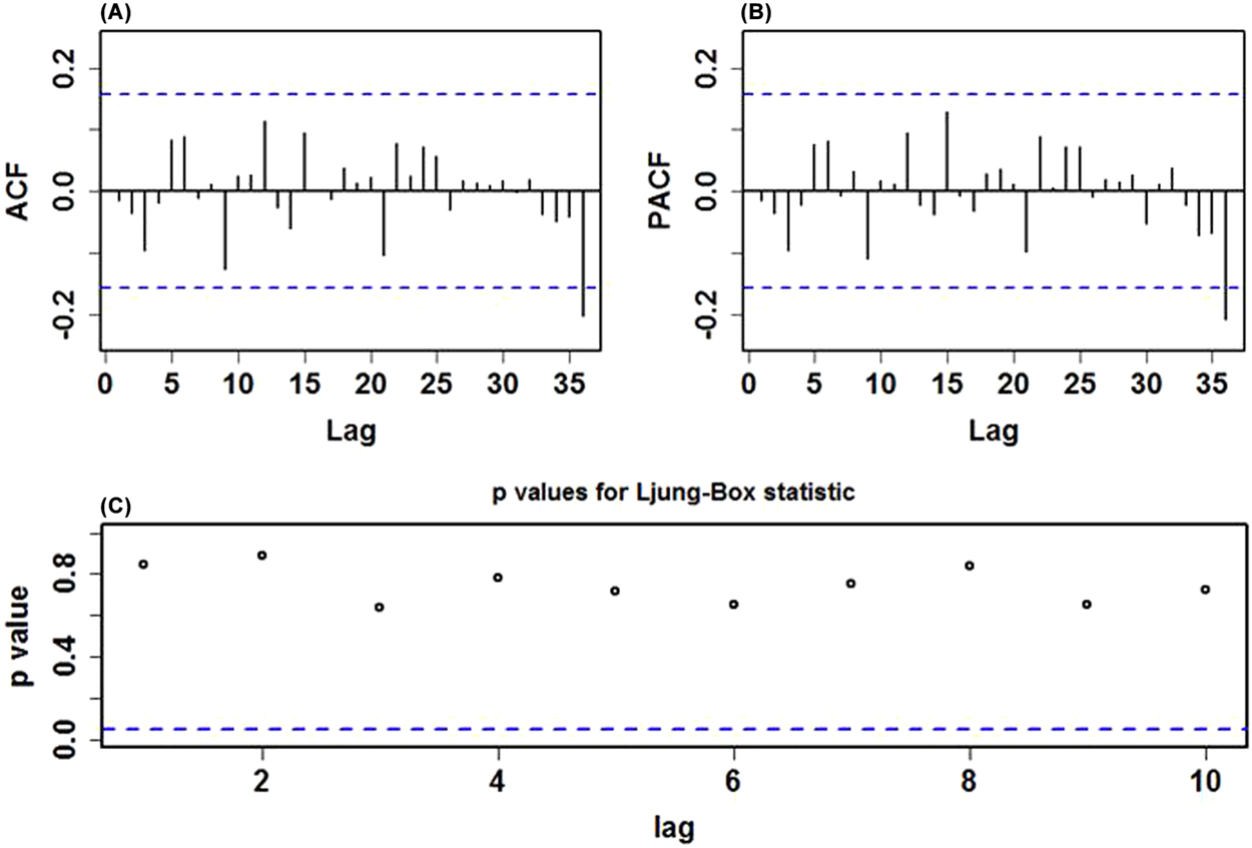
Diagnostic checking for the residual sequence generated by the SARIMA(0,1,3)(0,1,1)_12_ method. (**A**) ACF diagram; (**B**) PACF diagram; (**C**) Ljung-Box testing results. All of the correlation coefficients fell into the 95% uncertainty levels with the exception of the lag at 36. Accordingly, we believed that this preferred method can adequately model the HFRS series.

Before modeling the training samples from January 1, 2005 through December 31, 2018, the ADF test was applied to the data (ADF = − 3.621, *p* < 0.001), being indicative of a stationary series, which met the requirement of the SARIMA method establishment. However, it appeared that there was an unstable variance and mean in this series over time (Fig. 2B). Accordingly, the logarithmic and square root transformations were applied to the series to stabilize its variance, indicating a similar trend between these two series (Supplementary Fig. S3**)**. After an attempt, it seemed that the logarithmic transformation was more suitable for the SARIMA model construction. Subsequently, the seasonal and nonseasonal differences were performed to reduce its trend and seasonality of this processed series (Supplementary Figs S4-S6). Now, the transformation and differencing have made the data achieve completely stationary. Based on the ACF and PACF plots of this stationary series, several possible SARIMA methods were chosen (Table 1). Further, the results from the goodness of fit tests intimated that the SARIMA(0,1,3)(0,1,1)_12_ tended to be the best-fitting model, as this model had the lowest values of AIC = − 851.561 and BIC = − 836.344, together with the maximum value of Log-likelihood=430.781, and the parameters of this model indicated a significant difference at the 5% level (Table 2). Moreover, a greater *p*-value than 0.05 under the Ljung-Box test meant that the residual series successfully accomplished white noise (Fig. 3). In addition, the LM test indicated that the ARCH effects existed in the original observed data were also eliminated (Table 3). This optimal model passed all required checking, and thus can be utilized to perform projections for the future (Table 4). Likewise, following the modeling steps, we conducted a sensitivity analysis using the additional training subset from January 1, 2005 to December 31, 2017 to verify the model’s uncertainty. The obtained best-conducting SARIMA model and its goodness of fit testing results are summarized in Supplementary Tables S1-S3 and Fig. S7.

**Table 1:** Comparisons of the goodness of fit test for the five candidate SARIMA models.

Model	AIC	BIC	Log-Likelihood
SARIMA(0,1,3)(0,1,1)_12_	−851.561	−836.344	430.781
SARIMA(0,1,3)(1,1,0)_12_	−850.759	−835.542	430.380
SARIMA(0,1,3)(0,1,0)_12_	−841.185	−829.011	424.592
SARIMA(0,1,2)(0,1,1)_12_	−839.460	−827.287	423.730
SARIMA(1,1,1)(0,1,1)_12_	−840.981	−828.807	424.491

**Table 2:** Estimated parameters for the optimal SARIMA(0,1,3)(0,1,1)_12_ method and statistical test for them.

Parameter	Estimates	Standard error	*t*	*p*-value
MA1	0.171	0.079	2.156	0.033
MA2	0.215	0.080	2.683	0.008
MA3	0.344	0.078	4.394	<0.001
SMA1	0.434	0.081	5.362	<0.001

**Table 3:** ARCH tests for the original series and residual series from the optimal three methods.

Lags	Observed value		SARIMA model		SETAR model		LSTAR model	
	LM-test	*p*-value	LM-test	*p*-value	LM-test	*p*-value	LM-test	*p*-value
1	74.744	<0.001	6.246	0.012	2.174	0.140	0.514	0.473
3	112.870	<0.001	7.245	0.064	2.496	0.476	0.662	0.882
6	130.200	<0.001	8.536	0.201	1.547	0.956	1.363	0.968
9	132.260	<0.001	8.987	0.439	5.342	0.804	5.803	0.759
12	213.890	<0.001	14.800	0.253	16.330	0.177	49.191	<0.001
15	217.380	<0.001	12.800	0.612	22.205	0.103	58.949	<0.001
18	215.910	<0.001	14.922	0.667	23.448	0.174	29.115	0.070
21	214.690	<0.001	15.580	0.793	19.559	0.549	21.089	0.454
24	208.850	<0.001	16.361	0.875	21.034	0.637	18.968	0.754
27	210.920	<0.001	17.004	0.931	0.637	0.735	21.876	0.744
30	210.640	<0.001	17.276	0.969	25.556	0.698	24.128	0.766
33	208.650	<0.001	17.783	0.986	30.500	0.592	26.961	0.761
36	208.330	<0.001	23.074	0.953	38.099	0.374	34.014	0.563

**Table 4:** Comparisons between the actual values from January to September in 2019 and the forecasts from the optimal three methods.

Month	Actual value	SARIMA model	SETAR model	LSTAR model
January	0.079	0.072	0.065	0.065
February	0.051	0.043	0.046	0.044
March	0.053	0.070	0.049	0.045
April	0.050	0.073	0.064	0.065
May	0.067	0.094	0.085	0.093
June	0.072	0.090	0.073	0.081
July	0.050	0.069	0.053	0.054
August	0.029	0.046	0.044	0.039
September	0.025	0.046	0.042	0.041

### The best-performing regime-switching methods

**Figure 4:**
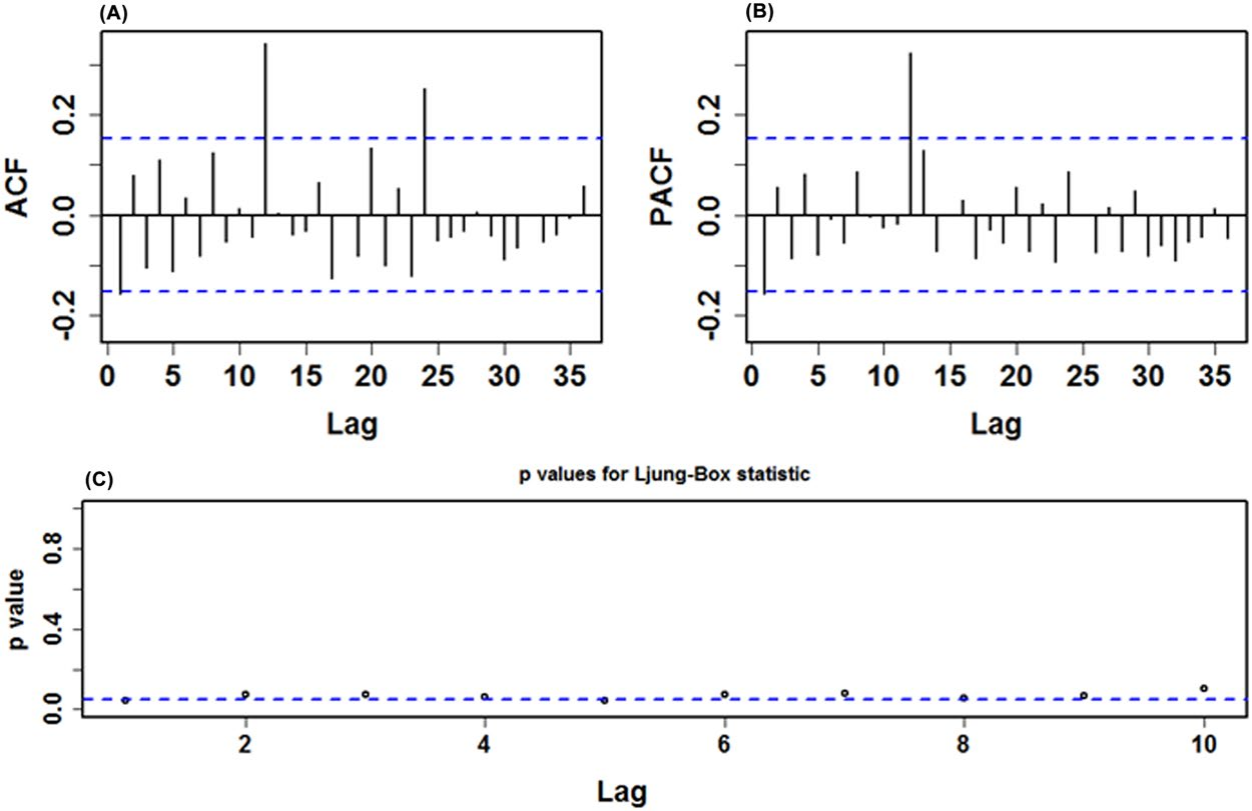
Diagnostic testing plots for the residual series from the best-fitting SETAR(2,4,5) method. (**A**) ACF diagram; (**B**) PACF diagram; (**C**) Ljung-Box testing results. None of the correlation coefficients were out of the 95% uncertainty limits except for the significant spikes at 12 and 24 in the ACF and at 12 in the PACF. Sometimes, it is also reasonable because the high-order correlations may readily exceed the 95% uncertainty limits by chance.

**Figure 5:**
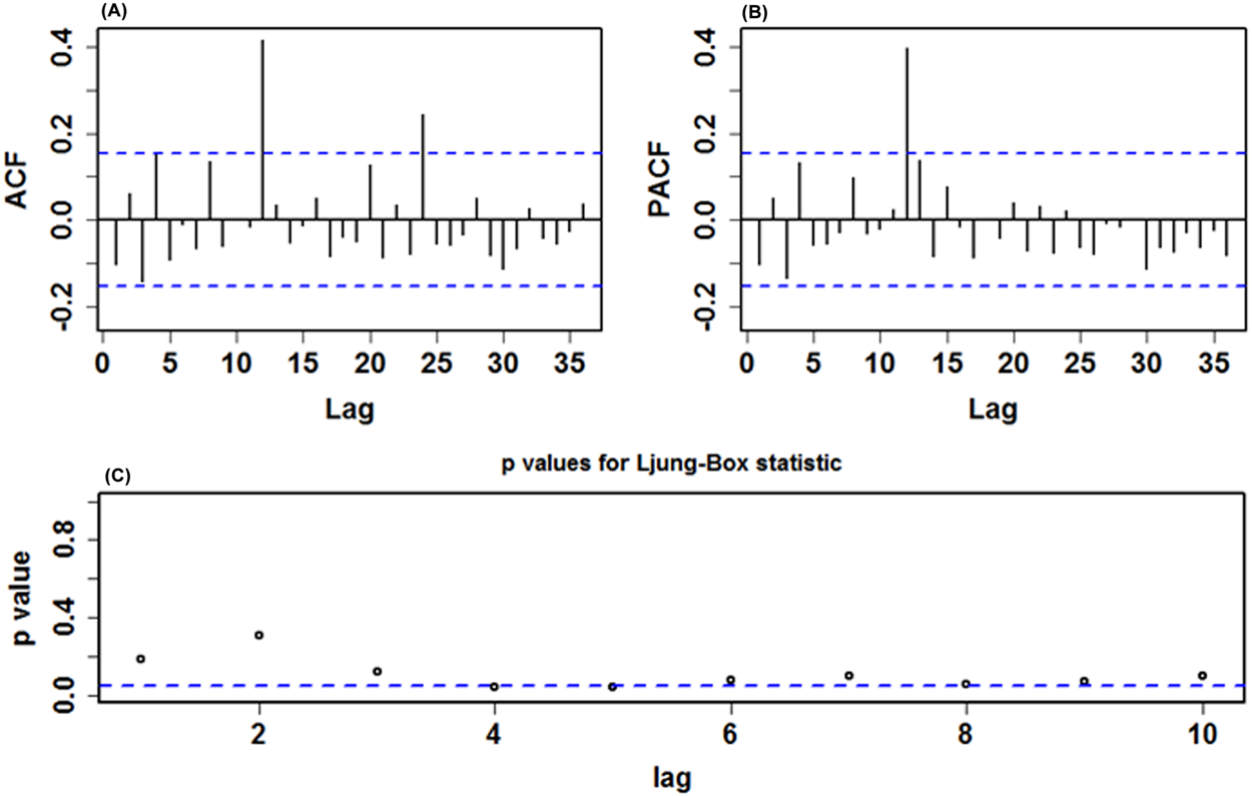
Statistical test plots for the residual series from the best-fitting LSTAR(2,4,5) method. (**A**) ACF diagram; (**B**) PACF diagram; (**C**) Ljung-Box testing results. No correlation coefficient other than the lags at 12 and 24 in the ACF and at 12 in the PACF lay outside the 95% uncertainty intervals.

The results of the BDS test are displayed in Table 5, all statistics revealed a *p*-value less than 0.05, being suggestive of a highly nonlinear mechanism of the data. Consequently, it is necessary to establish the model-based nonlinear SETAR and LSTAR methods fitted to the HFRS incidence series. In this work, we used the grid search to detect the appropriate parameters (d, *p*_*1*_, and *p*_2_) for these two methods. After trying over and over again, we found that the nominal AIC was smallest when the delay parameter *d* = 2 (*d* = 1, 2, 3, 4, and 5 corresponded to the nominal AIC = − 756.0, −852.4, −798.5, −753.3, and −781.0, respectively), and as shown in Supplementary Table S4, suggesting that the pooled AIC had the lowest value of −754.045 when *p*_1_ and *p*_2_ were 3 and 5, respectively, in the SETAR method, and yet the *p*_1_ = 4 and *p*_2_ = 5 were competitive. Thus, an approximation of these possible parameters of the SETAR method to the HFRS incidence series was attempted, the comparative results are given in Table 6, and the mimic performance measures of the SETAR(2,4,5) method provided smaller values than that of the SETAR(2,3,5) method. The results hinted that the SETAR(2,4,5) method seemed more suitable for our data (Supplementary Table S5), and the statistical checking results for the residuals from this method are shown in Table 3 **and** Fig. 4. Further, we tested the preferred three-regime SETAR method, which produced a poorer performance with MAPE = 21.820% than the best-fitting two-regime. Consequently, we selected the two-regime model as the optimal in our study. In the meantime, we could also get the best-fitting LSTAR(2,4,5) approach using the grid search (Table 6, Fig. 5 and Supplementary Tables S5-S6). Whereafter, the out-of-data forecasts can be made by using these two best-undertaking approaches (Table 4). Similarly, the preferred SETAR and LSTAR approaches used to account for the models’ uncertainty can be established based on the above-mentioned steps, and all results are listed in Supplementary Tables S7-S9 and Figs S8-S9.

**Table 5:** Resulting BDS testing results for the residuals of the optimal SARIMA(0,1,3)(0,1,1)_12_ method.

Epsilon	Dimension	Statistic	*p*-value
0.008	2	6.952	<0.001
0.008	3	7.844	<0.001
0.008	4	8.561	<0.001
0.008	5	9.194	<0.001
0.015	2	5.228	<0.001
0.015	3	5.350	<0.001
0.015	4	4.825	<0.001
0.015	5	4.351	<0.001
0.023	2	3.911	<0.001
0.023	3	4.208	<0.001
0.023	4	3.890	<0.001
0.023	5	3.539	<0.001
0.031	2	2.948	<0.001
0.031	3	3.247	0.001
0.031	4	3.135	0.002
0.031	5	2.600	0.009

**Table 6:** Comparisons of the mimic results from the plausible SETAR and LSTAR methods.

Model	MAE	MAPE	RMSE	MER
SETAR(2,3,5)	0.0135	0.1998	0.0179	0.1787
SETAR(2,4,5)	0.0134	0.1974	0.0178	0.1781
LSTAR(2,2,5)	0.0130	0.1840	0.0185	0.1728
LSTAR(2,4,5)	0.0129	0.1804	0.0184	0.1708

### Measuring for forecasting accuracy

**Figure 6:**
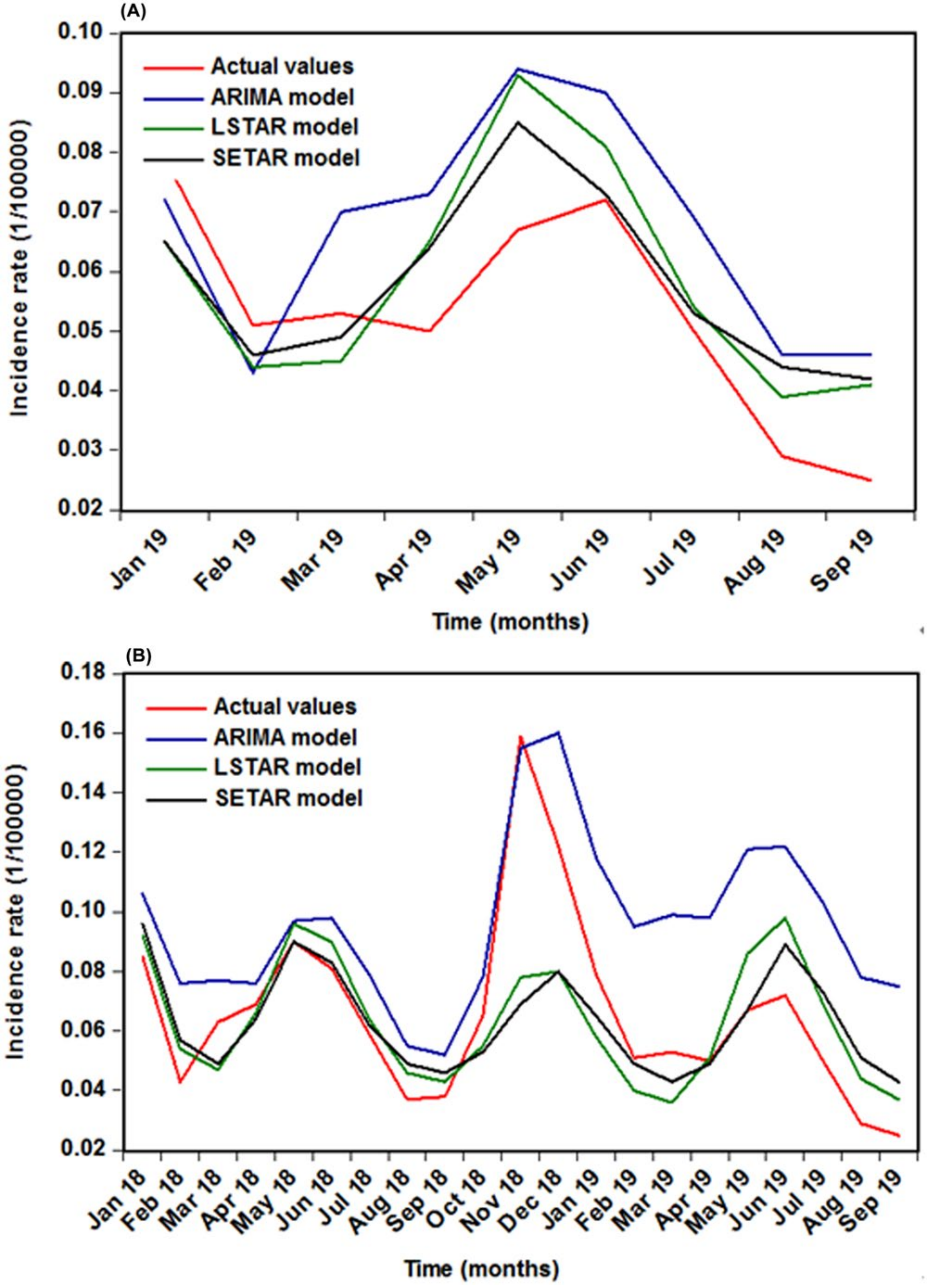
The multi-step-ahead predictions using the selected optimal three methods. (**A**) 9-data ahead forecasting; (**B**) 21-data ahead forecasting. As a whole, the SETAR and LSTAR approaches can better capture the epidemic trends of HFRS morbidity.

**Figure 7:**
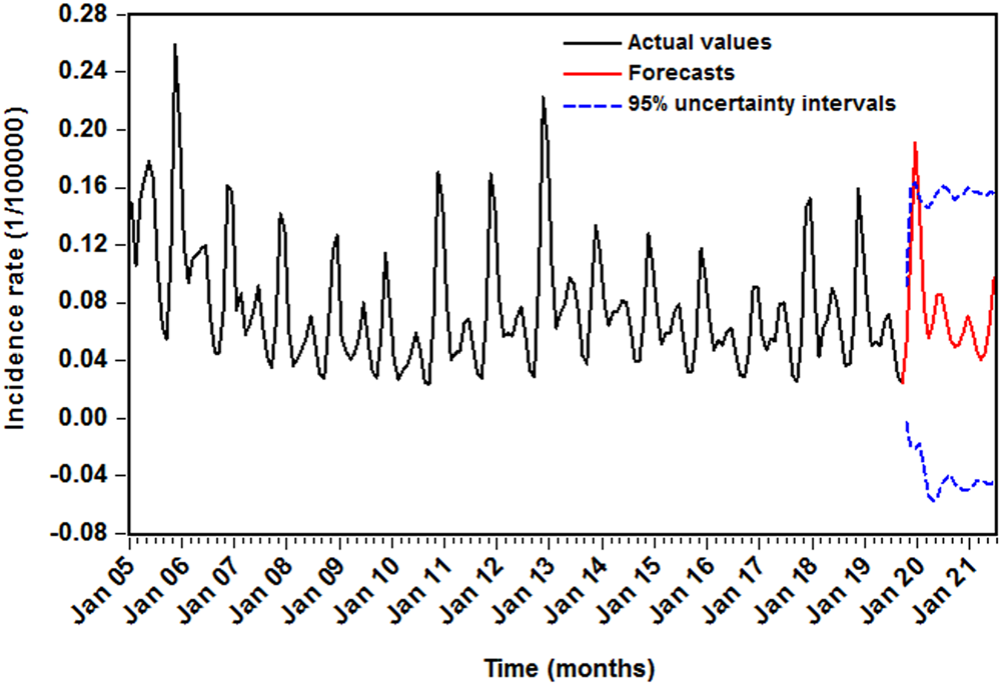
The predictive results from October 2019 to June 2021 and their 95% uncertainty bounds using the SETAR(2,3,5) method fitted to the entire data. As shown, the estimated value in December 2019 was outside the 95% uncertainty bounds, it seemed to show the possibility of an outbreak.

The comparative results of the out-of-sample forecasting are presented in Table 7. As can be seen from the data, the SETAR and LSTAR approaches visibly provided smaller values among the measures of MAE, MAPE, RMSE, and MER in both two forecasting sets, and the SETAR approach was slightly superior to the LSTAR method in view of the above four indices. Looking at Fig. 6, compared with the SARIMA model, also indicating that the SETAR and LSTAR methods could better capture the dynamic dependent structure of the data. In the light of these results, we thus constructed the SETAR model depending on the entire HFRS incidence data to undertake a projection into June 2021, and the 95% predictive intervals were resorted to simulation with 5,000 sizes (Fig. 7). According to the predictive results, it appeared that there would be a likelihood of HFRS outbreak in December 2019 since its forecast in this month was out of the 95% uncertainty intervals.

**Table 7:** Comparisons of the forecasting performances among the selected three methods.

Model	MAE	MAPE	RMSE	MER
**9-step-ahead forecasts**				
SARIMA	0.0174	0.3873	0.0185	0.3298
SETAR	0.0101	0.2412	0.0119	0.1912
LSRAR	0.0121	0.2604	0.0136	0.2290
**Percentage reductions (%)**				
SETAR vs. SARIMA	41.9540	37.7227	35.6757	42.0255
LSRAR vs. SARIMA	30.4598	32.7653	26.4865	30.5640
**21-step-ahead forecasts**				
SARIMA	0.0303	0.6062	0.3482	0.4585
SETAR	0.0152	0.2388	0.0246	0.2296
LSRAR	0.0163	0.2406	0.0236	0.2466
**Percentage reductions (%)**				
SETAR vs. SARIMA	49.8350	60.6071	92.9351	49.9237
LSRAR vs. SARIMA	46.2046	60.3101	93.2223	46.2159

## Discussion

Recently, the recurring risk of HFRS has been an increasing concern in China^9–13^. Forecasting based on high accuracy models may provide a useful aid in the development of a preventive and control system, as well as the reallocation of the limited resources. In this study, we established SETAR method and LSTAR approach to analyze and forecast the temporal tendencies of HFRS, moreover, the predictive abilities of the frequent use of SARIMA method and above used methods were compared. The time series analysis results demonstrated a valuable estimation for the 9-data-ahead (short-term) and the 21-data-ahead (long-term) predictions using the SETAR and LSTAR approaches, which provided more accurate and robust predictions for the HFRS morbidity series relative to the SARIMA approach, additionally, the SETAR model seemed to slightly overmatch the LSTAR model in the predictive power. Furthermore, given the MAPE value that is often used to measure the accuracy of a prediction^31^, suggesting no significant deterioration in the long-term prediction performance in comparison to that in the short-term predictions (the MAPE values were 0.2388 vs. 0.2412 in the SETAR method and 0.2406 vs. 0.2604 in the LSTAR method). Our investigation meant that the predictive performances of these two methods maintained robustness, and they can be recommended as a useful tool in understanding and predicting the epidemic patterns of HFRS, which will be of fundamental importance for the prevention and control of this disease. What’s more, we observed that the SARIMA method showed an unacceptable level of accuracy with the predictive period increased, which further confirmed that the SARIMA approach is suited to evaluate the short-term temporal levels of a time series^16^.

The SARIMA method assumes that there exists certain linear link between the future epidemic trajectories of the target time series and the changing state of its historical data^32^, thus, it has been emerged as the most popular model to perform a forecast for the future by considering the overall trends and seasonal pattern of a time series with seasonality or non-seasonality^16,32^. For example, Cong *et al*. built a SARIMA(1,0,0)(0,1,1)_12_ method to forecast the epidemic patterns of influenza incidence in China^16^. Fu *et al*. developed a SARIMA(0,0,2)(0,1,1)_12_ method to conduct a forecast for the incidence series of hand-foot-mouth disease in Zhejiang Province^33^. Albeit this method frequently provides a good approximation to the target time series, it suffers from weaknesses in handling nonlinear patterns and which is only suitable for undertaking short-term forecasting^16,17^. All in all, this is in agreement with our findings. In the real world, the development and occurrence of diseases are associated with many drivers, which make the relationship between the observations show nonlinear modes. Therefore, it is necessary to test the data-generating mechanism prior to establishing a model for the target time series. In this study, the BDS test was applied to the residuals produced by the SARIMA method to detect nonlinearity of the HFRS incidence series, suggesting a notable nonlinear tendency. In this context, nonlinear methods are seemingly appropriate. To our best knowledge, the model-based nonlinear SETAR and LSTAR approaches were the first time employed to predict HFRS incidence in the forecasting domain of infectious diseases, and our experimental results also demonstrated their usefulness in the prediction for the HFRS morbidity series. However, for one thing, considering the different types of non-linear modes in data-generating process^18^, further investigations are required to evaluate the suitability for forecasting other infectious diseases; for another, other nonlinear statistical models (including ANNS^19^, ARCH^21^, SVM^20^, etc.) have also been established to study the temporal behaviors of infectious diseases. Collectively, further comparisons between our used methods and the above discussed models are also required in order to find the most accurate one that captures the nonlinear relationship. Besides, also notice that mathematical epidemiology has played an important role in the understanding of infectious disease transmission in human populations in the past decades^34^. Among which, the mechanistic models based on time series (such as susceptible exposed infectious recovered (SEIR) model or SIR model) have widely been used to model the transmission dynamics of contagious diseases such as measles^35^, coronavirus disease 2019^36^, dengue fever^37^, HFRS^2^, etc, in that the SEIR or SIR model can easily explain inhomogeneous mixing in a phenomenological manner by considering the nonlinear dependence of contact rates between susceptible population and hosts^38^, and thus can be used to assess the key parameters of infectious processes and clarify the potential processes driving the transmission dynamics of infectious diseases^39^. However, the SEIR or SIR is a deterministic model with the assumption that the infectious persons are independently and randomly mixing with all other persons^2^. As mentioned above, in practice, the transmission of contagious diseases is limited and affected by varying indeterminate divers (e.g., climatic variability, seasonal variation, variations in pathogens, or government policy)^2,40,41^. Under such circumstances that the morbidity data are often inclined to show uncertainty and nonlinearity^32^, the SEIR or SIR model may obtain unsatisfactory forecasting results. At this time, our used regime-switching methods may be more suitable and more convincing, because these nonlinear models assign multiple potential drivers and comprehensive effects of uncertainty factors that may drive the disease occurrence and development to a univariate time series, and then performing prediction by identifying the potential relationships between the future state of the incidence series and the past and present internal rules of the historical series. Moreover, the regime-switching methods with the advantages of low-cost data collection and extensive application in practice are easy to develop (based only on intrinsic variables) and can obtain relatively satisfactory predictive accuracy as evidenced by our experiment results. Despite these advantages of the regime-switching models, much work is still required to compare the real forecasting effects between mechanistic models and regime-switching models.

Our research manifested a downward trend in HFRS morbidity in the whole study period, which is similar to that observed in some countries in Asia^42^. This may mainly be attributed to the government’s continued efforts such as the implementation of a series of rodents’ control measures, the improvement of living standards, the increased urbanization and farm mechanization, and the development of the targeted vaccine and so forth^7^. Under these efforts, some achievements have been attained, but we observed that the epidemic trend started to rise since 2016, which is seemingly not until December 2019 that such an increase will reach the climax with the highest incidence of 0.191 per 100,000 people according to our predictions using the SETAR method, and there may be a risk of outbreak. Regarding the substantial increase in HRFS morbidity, one plausible explanation may be related to the effects of climatic change which has posed a serious threat on the global scale^1,43^. HFRS has been identified as a climate-sensitive disease because weather variability has a direct or indirect impact on the rodent population dynamics, such as reproductive rates and incubation period, crop output that serves as the foremost food sources for rodents, and viral exposure opportunities in predisposed population^1,11,43^; another main reason may emanate from the fact that periodic outbreak is among the most important epidemiological characteristics of HFRS^44^. Previous work has reported a natural cyclical pattern in HFRS morbidity with around 7–12 years^44,45^, this phenomenon was also observed in our work, despite with a periodic outbreak being 3–5 years. Besides, new Hantavirus subtypes may also be associated with this sudden increase since a recent study has shown that the emergence of new Seoul viruses raises new challenges to fight against HFRS^11^. Also, investigations into other plausible causes still go on.

Understanding the seasonal distribution of infectious diseases is of great significance for the analysis and estimation of the diseases’ transmission patterns. Our analytical results exhibited a strong seasonality in HFRS morbidity with a dual-peak pattern, where a strong peak was observed in November until January in the next year and a weak one in May and June per year. The observation fits well with that reported in most areas of China, such as Qingdao^11^, Zibo^46^, Zhejiang^47^, Changsha^45^, Heilongjiang^4^, Shenyang^7^, Hubei^44^, Liaoning and Anhui^43^, and also concurs well in Korea^48^, but inconsistent with that reported in Guangzhou (which peaked in February until May)^12^. Such a significant difference in seasonal behaviors is predominantly responsive to climatic and demographic factors in the northern hemisphere city, Guangzhou, and its climate is characterized by a wet of high temperatures and a high humidity index, which is significantly different from other areas in China^12^. In China, the double peak activities in HRFS morbidity may be mainly reeling from its etiologic factors and climatic factors^2,10,11,44^. Earlier work has found that the HTNV-related HFRS infections are reported all through the year, yet most of them occur in fall and winter whereas the SEOV-related cases are typically observed in spring, and these two pathogenic agents are predominantly spread by A. agrarius and R. norvegicus rodents, respectively^10,44^. Significantly, climatic drivers, such as temperature, relative humidity, precipitation, etc., can affect hosts’ reproduction and thus causing the transmission of HFRS^2,11,44^. For instance, the relationship between temperature and relative humidity and HFRS epidemic exhibits a U-shaped curve^11^, which is in agreement with the peak activities present in winter and summer in HFRS incidence.

This study focused on an investigation into the suitability for application in analyzing and forecasting the epidemic trends in HFRS morbidity using the SETAR and LSTAR methods and has shown their usefulness. Nevertheless, several potential shortfalls need to be considered. Firstly, the under-reporting and under-diagnosis may still be inevitable, in spite of the well-monitored data quality regarding infectious diseases in China, Secondly, we only collected the monthly and yearly HFRS cases absent from some detailed information (such as age, sex, and occupation) due to their unavailability, which precludes further stratified or sensitivity analysis that accounts for the models’ uncertainty. Thirdly, whether these methods are applicable to study HFRS epidemic in other areas needs to be further verified. Finally, in application, these methods entail to be updated with the newly aggregated data in order to maintain their high prediction accuracies.

In conclusion, our findings suggested that the SETAR and LSTAR methods showed superiorities in tracking the temporal patterns than the most commonly adopted SARIMA approach, moreover, they can undertake long-term forecasting, which can function as a useful tool in offering an advanced warning for the epidemiological characteristics of HRFS, and therefore formulating a long-term targeted prevention and control plans in response to this threat of HRFS. Additionally, China is still afflicted with the risk of HFRS outbreak under the present control and prevention strategies. Consequently, more effective control measures are warranted.

## Supplementary information

Supplementary Information.

## Data Availability

All data were presented in our analytical results or please contact the first author or the corresponding author on reasonable request.
