# Supplementary material for: Time series analysis of temporal trends in hemorrhagic fever with renal syndrome morbidity rate in China from 2005 to 2019
Source: Sci Rep. 2020 Jun 15;10:9609. doi: 10.1038/s41598-020-66758-4 (PMC7295973; doi:10.1038/s41598-020-66758-4)
Supplement: Supplementary file 1 — Supplementary Information. [file 41598_2020_66758_MOESM1_ESM.pdf]

Supplementary information: Time series analysis of temporal trends in hemorrhagic fever with renal syndrome morbidity rate in China from 2005 to 2019

Yongbin Wang[1,+], Chunjie Xu[2,+], Weidong Wu[1], Jingchao Ren[1], Yuchun Li[1], Lihui Gui[1] & Sanqiao Yao[1]

[1] Department of Epidemiology and Health Statistics, School of Public Health, Xinxiang Medical University, Xinxiang, Henan Province, 453003, P.R. China

[2] Department of Occupational and Environmental Health, School of Public Health, Capital Medical University, Beijing 100069, P.R. China

* Corresponding author: Yongbin Wang (wybwho@163.com), No. 601 Jinsui road, Hongqi district, Xinxiang city, Henan province, 453003, P.R. China; Tel: +86-373-3831646

[+] These authors contributed equally to this research

Supplementary Figure and Table captions

Figure S1. Time series plot measuring the yearly epidemic trends of HFRS. It appeared that there was a periodic change pattern with 3-5 years.

Figure S2. Decomposed seasonal factors for the HFRS morbidity series using the additive decomposition method. As displayed, the peak activities of HFRS morbidity mainly occurred in January, May, June, November and December per year, while the tough was observed in August and September every year.

Figure S3. Time series plots showing the HFRS morbidity sequence of the logarithmic and square root transformations. (A) Actual series; (B) log-transformed series; (C) square-root transformed series. It appeared that there was a similar trend in these two transformed series.

Figure S4. Autocorrelation function (ACF) and partial autocorrelation function (PACF) plots for the HFRS morbidity series from January to December 2018. As shown, a differenced method is required owing to the local maximum spikes occurring at lags 1, 12, 24 and 36 in the ACF graph.

Figure S5. Autocorrelation function (ACF) and partial autocorrelation function (PACF) plots for the seasonally differenced HFRS series. (A) The differenced HFRS series; (B) ACF diagram; (C) PACF diagram. It seemed that this differenced series was still non-stationary.

Figure S6. Autocorrelation function (ACF) and partial autocorrelation function (PACF) plots for the non-seasonally differenced HFRS series after a seasonal difference. (A) The differenced HFRS series; (B) ACF diagram; (C) PACF diagram.

This differenced series appeared to be stationary. Since there was a significant spike at lag 3 in the ACF, seemingly indicating an MA(3) process.

Figure S7. Diagnostic checking for the residual sequence generated by the optimal SARIMA(0,1,3)(1,1,0)$_{12}$ method. (A) ACF diagram; (B) PACF diagram; (C) Ljung-Box testing results. No correlation coefficient out of the 95% uncertainty bounds other than the lag at 36 in the ACF and PACF was observed. As a result, we confirmed that this preferred method can adequately model the HFRS series.

Figure S8. Diagnostic testing plots for the residual series from the best-fitting SETAR(2,3,5) method. (A) ACF diagram; (B) PACF diagram; (C) Ljung-Box testing results. None of the correlation coefficients were out of the 95% uncertainty limits except for the significant spikes at 12 and 24 in the ACF and at 12 in the PACF.

Figure S9. Statistical test plots for the residual series from the best-fitting LSTAR(3,5) method. (A) ACF diagram; (B) PACF diagram; (C) Ljung-Box testing results. No correlation coefficient other than the lags at 12 and 24 in the ACF and at 12 in the PACF lay outside the 95% uncertainty intervals.

Table S1. Estimated parameters for the optimal SARIMA(0,1,3)(1,1,0)$_{12}$ method and statistical test for them.

Table S2. ARCH tests for the original series from January 2005 to December 2017 and the residual series from the chosen optimal three methods.

Table S3. Comparisons between the actual values from January 2018 to September 2019 and the forecasts from the optimal three methods.

Table S4. Results of the grid search for 1 threshold.

Table S5. Results of the grid search for the possible LSTAR methods.

Table S6. Resulting BDS testing results for the residuals of the optimal SARIMA(0,1,3)(1,1,0)$_{12}$ method.

Table S7. Results of the grid search for 1 threshold based on the data from January 2005 to December 2017.

Table S8. Results of the grid search for the possible LSTAR methods based on the data from January 2005 to December 2017.

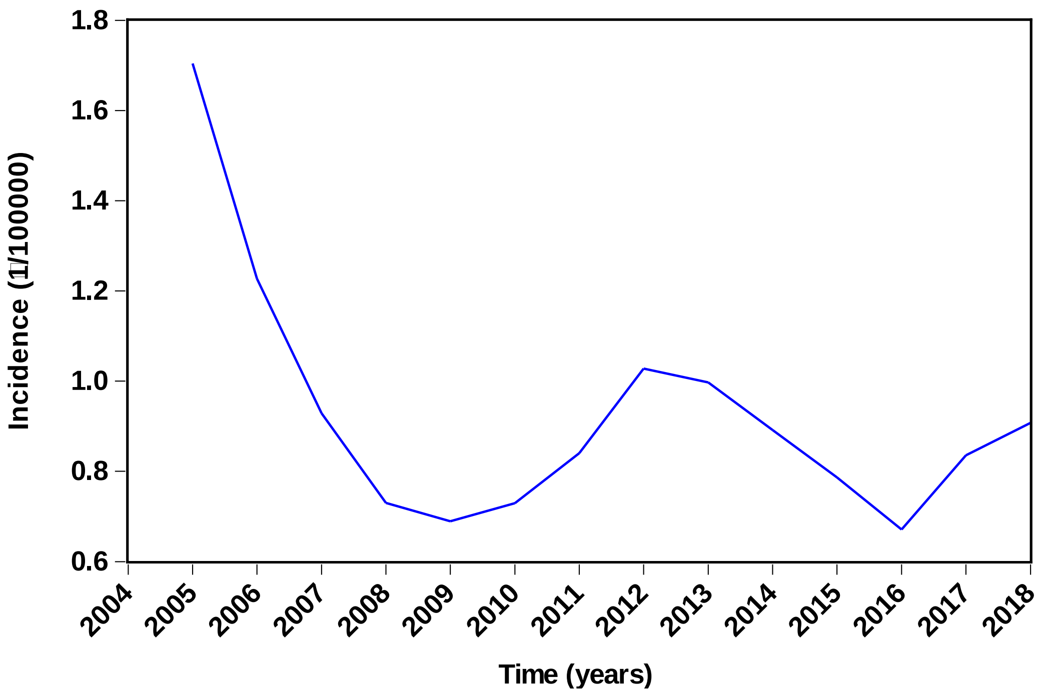

Figure S1. Time series plot measuring the yearly epidemic trends of HFRS. It appeared that there was a periodic change pattern with 3-5 years.

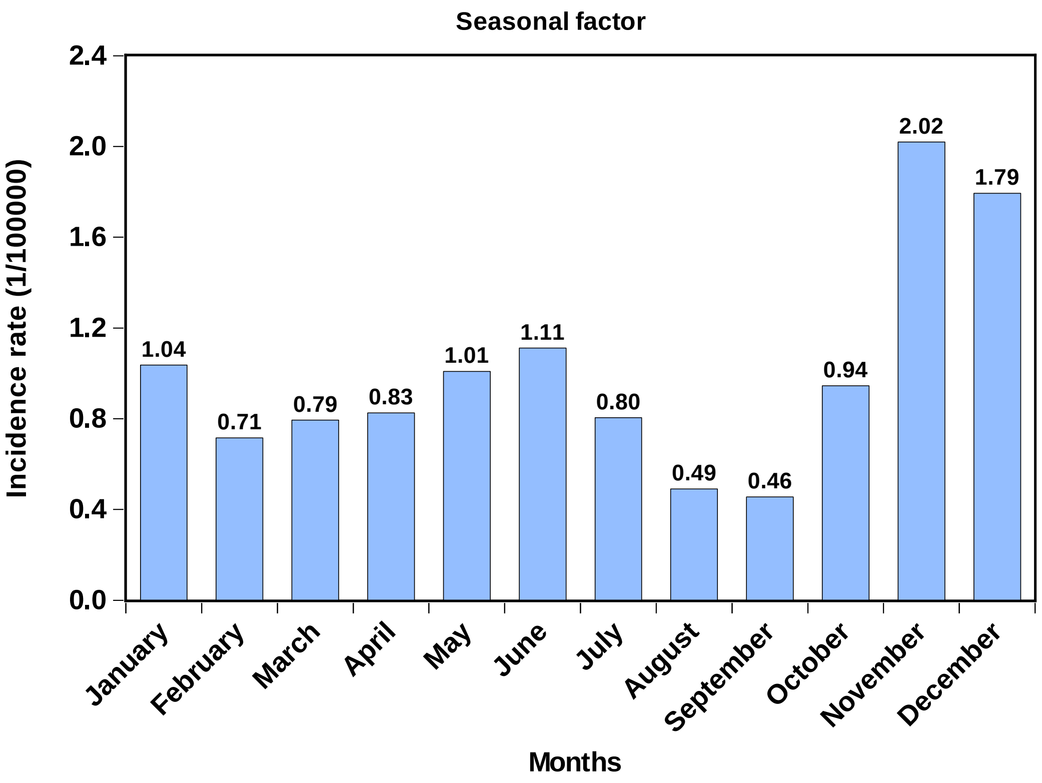

Figure S2. Decomposed seasonal factors for the HFRS morbidity series using the additive decomposition method. As displayed, the peak activities of HFRS morbidity mainly occurred in January, May, June, November and December per year, while the tough was observed in August and September every year.

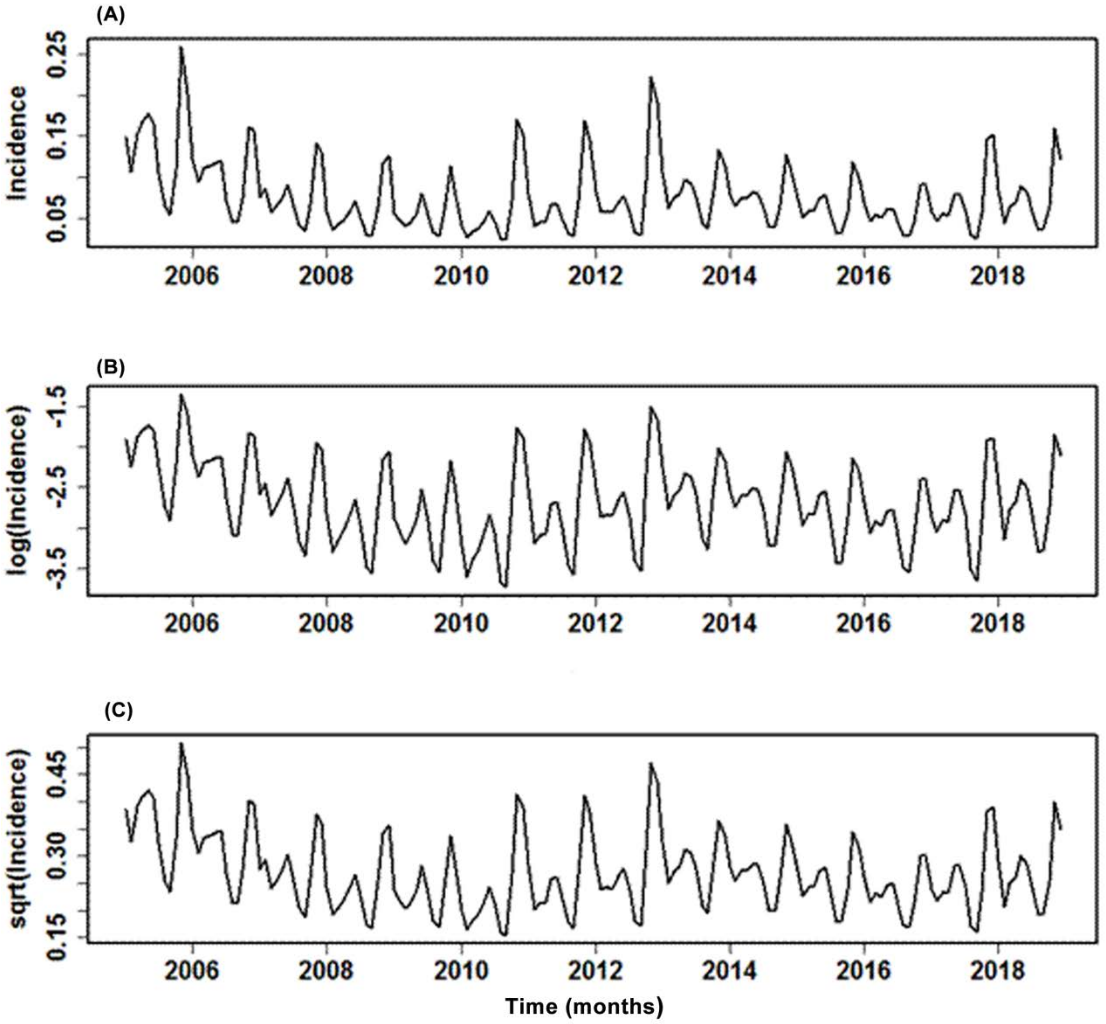

Figure S3. Time series plots showing the HFRS morbidity sequence of the logarithmic and square root transformations. (A) Actual series; (B) log-transformed series; (C) square-root transformed series. It appeared that there was a similar trend in these two transformed series.

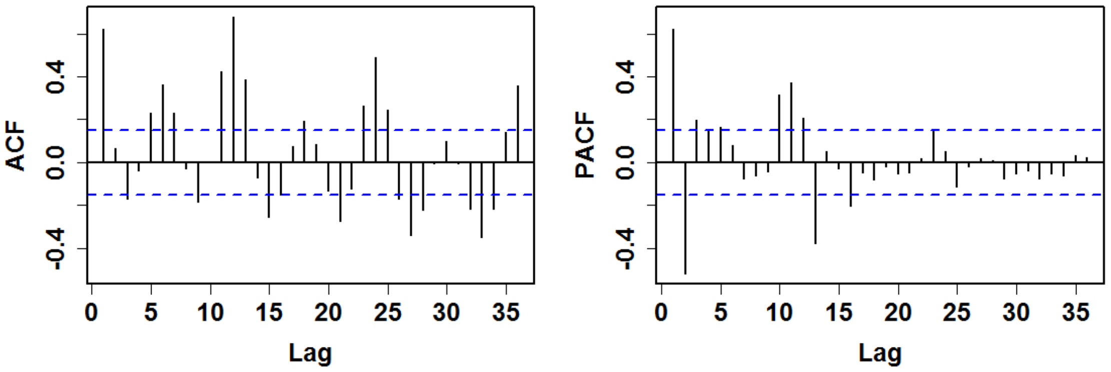

Figure S4. Autocorrelation function (ACF) and partial autocorrelation function (PACF) plots for the HFRS morbidity series from January to December 2018. As shown, a differenced method is required owing to the local maximum spikes occurring at lags 1, 12, 24 and 36 in the ACF graph.

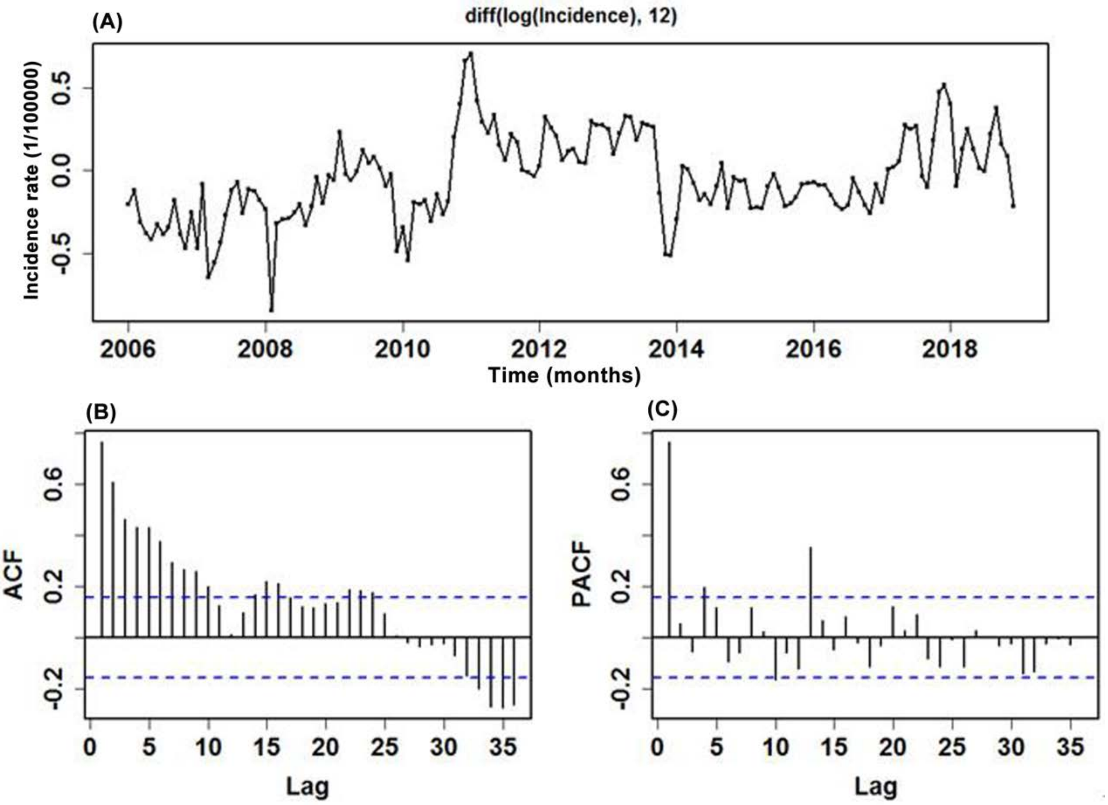

Figure S5. Autocorrelation function (ACF) and partial autocorrelation function (PACF) plots for the seasonally differenced HFRS series. (A) The differenced HFRS series; (B) ACF diagram; (C) PACF diagram. It seemed that this differenced series was still non-stationary.

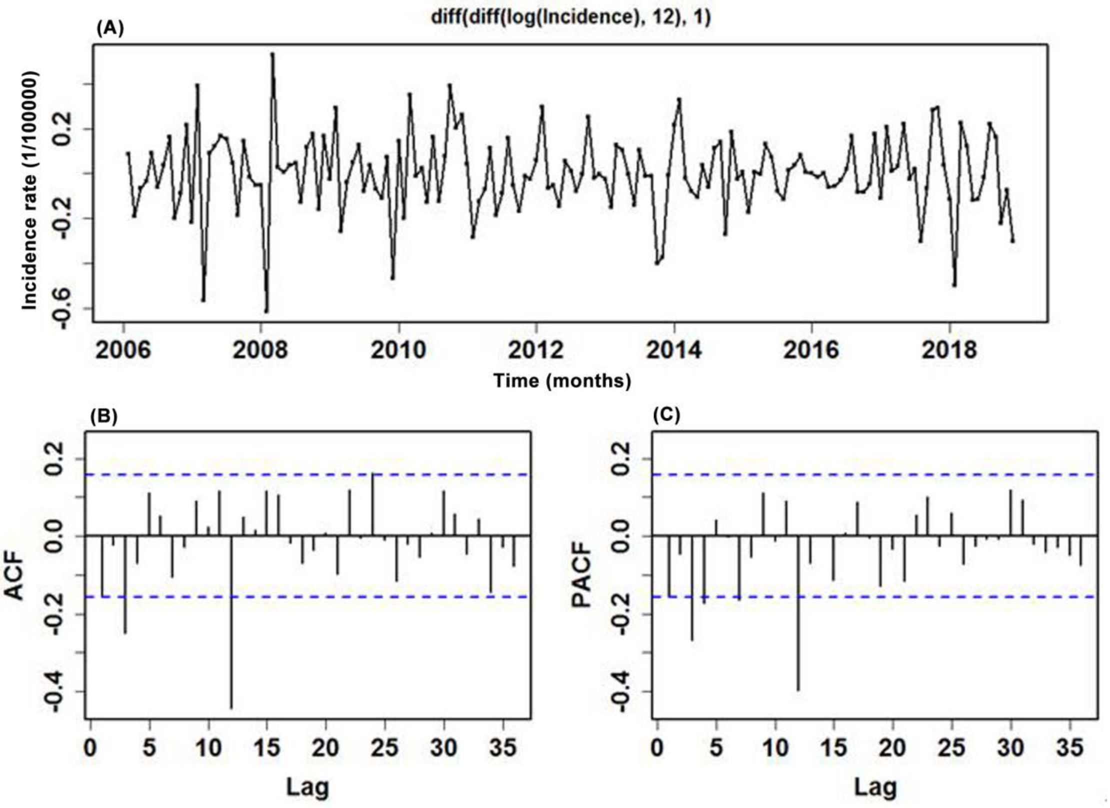

Figure S6. Autocorrelation function (ACF) and partial autocorrelation function (PACF) plots for the non-seasonally differenced HFRS series after a seasonal difference. (A) The differenced HFRS series; (B) ACF diagram; (C) PACF diagram. This differenced series appeared to be stationary. Since there was a significant spike at lag 3 in the ACF, seemingly indicating an MA(3) process.

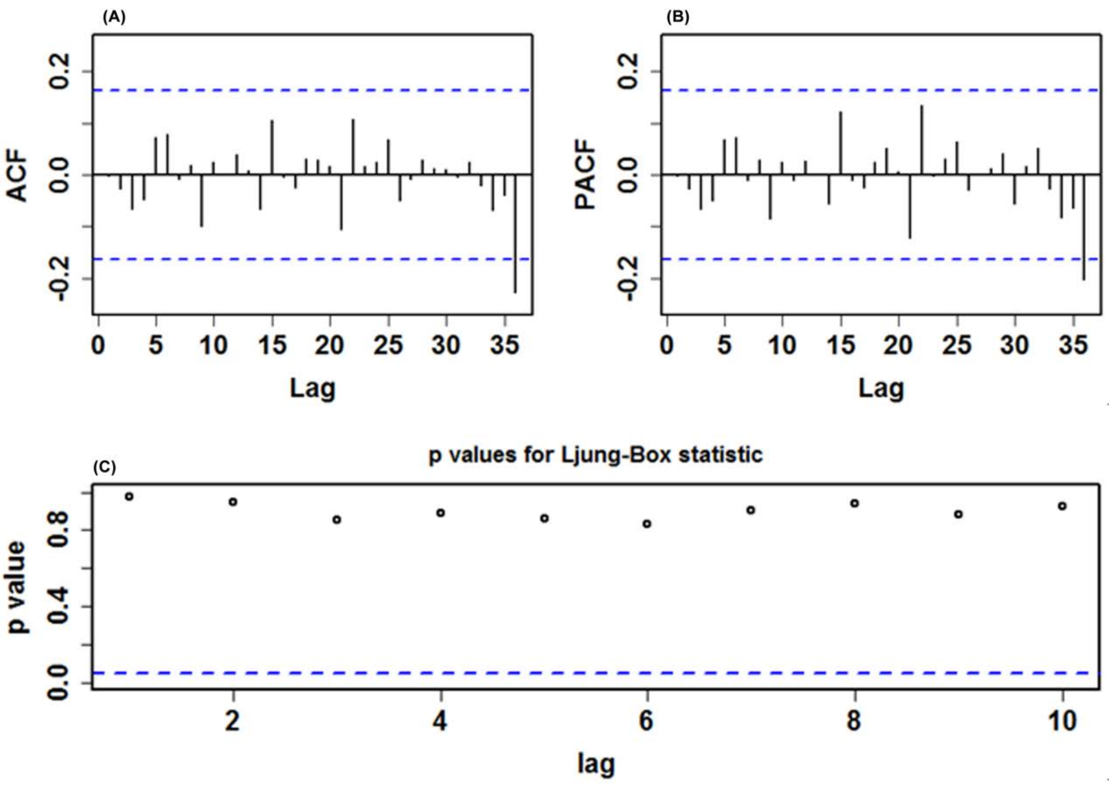

Figure S7. Diagnostic checking for the residual sequence generated by the optimal SARIMA(0,1,3)(1,1,0)$_{12}$ method. (A) ACF diagram; (B) PACF diagram; (C) Ljung-Box testing results. No correlation coefficient out of the 95% uncertainty bounds other than the lag at 36 in the ACF and PACF was observed. As a result, we confirmed that this preferred method can adequately model the HFRS series.

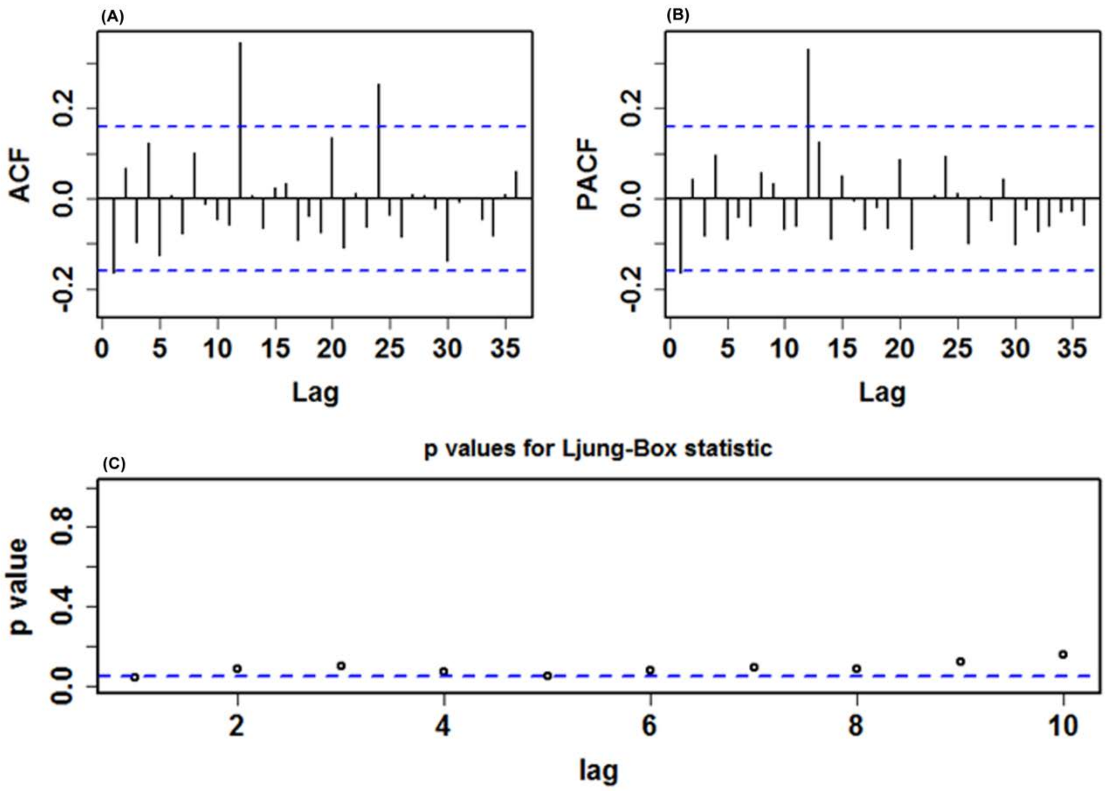

Figure S8. Diagnostic testing plots for the residual series from the best-fitting SETAR(2,3,5) method. (A) ACF diagram; (B) PACF diagram; (C) Ljung-Box testing results. None of the correlation coefficients were out of the 95% uncertainty limits except for the significant spikes at 12 and 24 in the ACF and at 12 in the PACF.

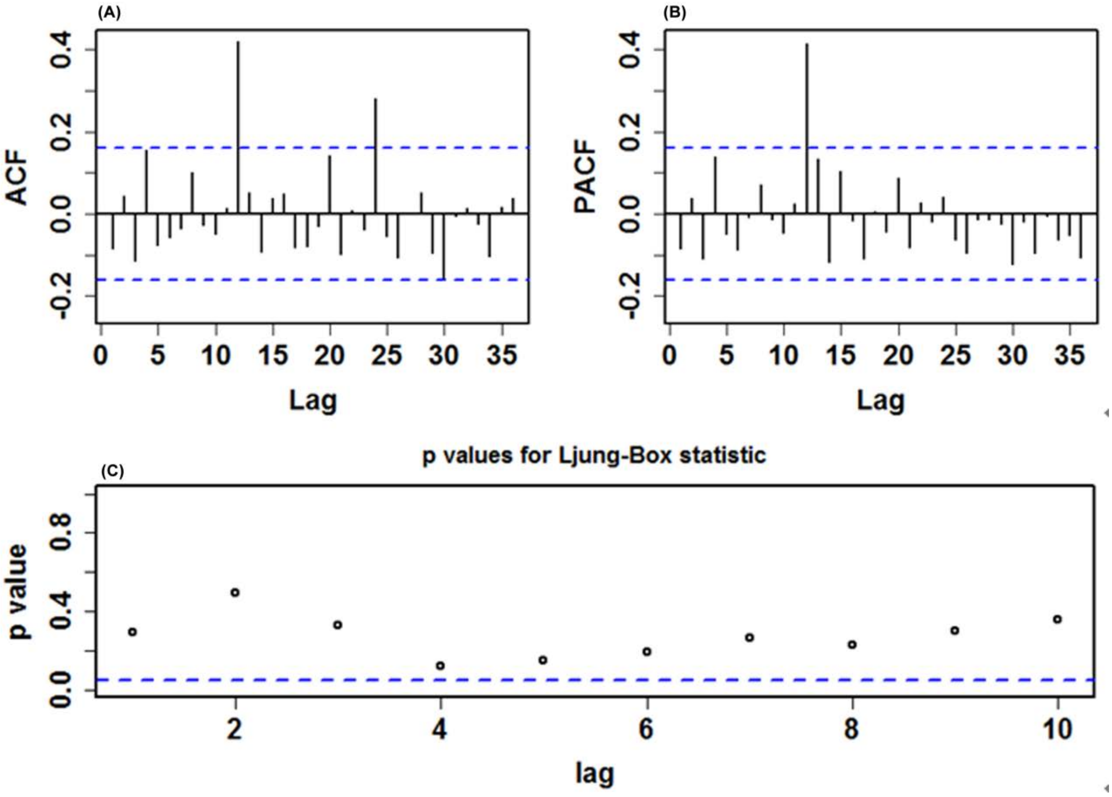

Figure S9. Statistical test plots for the residual series from the best-fitting LSTAR(2,3,5) method. (A) ACF diagram; (B) PACF diagram; (C) Ljung-Box testing results. No correlation coefficient other than the lags at 12 and 24 in the ACF and at 12 in the PACF lay outside the 95% uncertainty intervals.

Parameter	Estimates	Standard error	*t*	*p*-value
MA1	0.166	0.081	2.061	0.041
MA2	0.191	0.082	2.342	0.021
MA3	0.363	0.082	4.440	<0.001
SAR1	-0.307	0.083	-3.687	<0.001

Table S1 Estimated parameters for the optimal SARIMA(0,1,3)(1,1,0)$_{12}$ method and statistical test for them

Lags	Observed value		SARIMA model		SETAR model		LSTAR model	
	LM-test	*p*-value	LM-test	*p*-value	LM-test	*p*-value	LM-test	*p*-value
1	50.376	<0.001	3.489	0.062	3.521	0.061	0.432	0.511
3	66.775	<0.001	3.820	0.282	3.875	0.275	0.497	0.920
6	65.016	<0.001	4.544	0.715	1.661	0.948	2.831	0.830
9	64.101	<0.001	4.658	0.863	4.788	0.852	4.729	0.857
12	97.909	<0.001	5.468	0.941	17.164	0.144	49.678	<0.001
15	103.390	<0.001	4.685	0.995	21.327	0.127	60.064	<0.001
18	102.450	<0.001	5.776	0.997	15.272	0.643	32.914	0.017
21	100.780	<0.001	6.662	0.999	14.454	0.849	24.962	0.249
24	100.050	<0.001	6.570	1.000	13.770	0.952	24.219	0.449
27	100.220	<0.001	7.281	1.000	14.894	0.971	25.488	0.547
30	98.437	<0.001	8.052	1.000	17.816	0.961	32.249	0.356
33	96.569	<0.001	8.896	1.000	21.462	0.939	35.515	0.351
36	94.462	<0.001	17.687	0.996	27.190	0.855	39.760	0.306

Table S2 ARCH tests for the original series from January 2005 to December 2017 and the residual series from the chosen optimal three methods

Month	Actual values	SARIMA	SETAR(2,3,5)	LSTAR(2,3,5)
January-2018	0.085	0.106	0.096	0.092
February-2018	0.043	0.076	0.057	0.054
March-2018	0.063	0.077	0.049	0.047
April-2018	0.069	0.076	0.064	0.066
May-2018	0.090	0.097	0.090	0.096
June-2018	0.081	0.098	0.083	0.090
July-2018	0.059	0.079	0.062	0.064
August-2018	0.037	0.055	0.049	0.046
September-2018	0.038	0.052	0.046	0.043
October-2018	0.065	0.078	0.053	0.055
November-2018	0.159	0.155	0.069	0.078
December-2018	0.122	0.160	0.080	0.080
January-2019	0.079	0.118	0.065	0.058
February-2019	0.051	0.095	0.049	0.040
March-2019	0.053	0.099	0.043	0.036
April-2019	0.050	0.098	0.049	0.051
May-2019	0.067	0.121	0.067	0.086
June-2019	0.072	0.122	0.089	0.098
July-2019	0.050	0.103	0.073	0.069
August-2019	0.029	0.078	0.051	0.044
September-2019	0.025	0.075	0.043	0.037

Table S3 Comparisons between the actual values from January 2018 to September 2019 and the forecasts from the optimal three methods

Number	p_1	p_2	th	Pooled-AIC
1	3	5	0.110	-754.045
2	3	5	0.111	-752.349
3	4	5	0.110	-752.268
4	3	5	0.114	-751.700
5	5	5	0.110	-751.272
6	3	3	0.054	-751.134
7	3	3	0.052	-750.918
8	4	5	0.111	-750.422
9	4	5	0.114	-749.803
10	3	3	0.054	-749.507

Table S4 Results of the grid search for 1 threshold

The representation of the best-fitting SETAR(2,4,5)
$Y_t = \begin{cases} 0.057 + 2.331Y_{t-1} - 2.975Y_{t-2} + 0.699Y_{t-3} - 0.135Y_{t-4} + 0.025\varepsilon_1 & Y_{t-2} \le 0.054 \\ 0.004 + 0.902Y_{t-1} - 0.418Y_{t-2} + 0.181Y_{t-3} - 0.013Y_{t-4} + 0.175Y_{t-5} + 0.014\varepsilon_2 & Y_{t-2} > 0.054 \end{cases}$
The representation of the best-fitting LSTAR(2,4,5)
$Y_t = (0.047 + 3.047Y_{t-1} - 2.858Y_{t-2} + 1.201Y_{t-3} - 0.580Y_{t-4})(1 - G(Y_{t-4},100,0.04))$ $+ (-0.052 - 2.204Y_{t-1} + 2.533Y_{t-2} - 1.038Y_{t-3} + 0.576Y_{t-4} + 0.229Y_{t-5})G(Y_{t-4},100,0.04) + \varepsilon_1$

Table S5 The representations of the best-fitting SETAR and LSTAR methods.

Number	p_1	p_2	AIC	BIC	th	gamma
1	2	5	-1323.279	-1288.915	0.045	124.005
2	4	5	-1322.462	-1281.850	0.043	124.005
3	3	5	-1321.742	-1284.255	0.045	124.005
4	5	4	-1321.126	-1280.515	0.043	124.005
5	5	5	-1320.749	-1277.014	0.041	124.005
6	1	5	-1320.696	-1289.457	0.037	124.005
7	5	2	-1319.528	-1285.164	0.036	74.587
8	5	1	-1318.602	-1287.362	0.024	79.692
9	5	3	-1317.536	-1280.049	0.036	74.587
10	2	4	-1308.437	-1277.197	0.046	124.005

118 **Table S6** Results of the grid search for the possible LSTAR methods

Epsilon	Dimension	Statistic	*p*-value
0.008	2	5.214	<0.001
0.008	3	5.524	<0.001
0.008	4	6.203	<0.001
0.008	5	6.432	<0.001
0.015	2	4.937	<0.001
0.015	3	5.098	<0.001
0.015	4	4.663	<0.001
0.015	5	4.485	<0.001
0.023	2	3.441	0.001
0.023	3	3.842	<0.001
0.023	4	3.202	0.001
0.023	5	2.896	0.004
0.031	2	2.930	0.003
0.031	3	3.147	0.002
0.031	4	2.608	0.009
0.031	5	2.271	0.023

Table S7 Resulting BDS testing results for the residuals of the optimal SARIMA(0,1,3)$(1,1,0)_{12}$ method

Number	p_1	p_2	th	Pooled-AIC
1	3	3	0.054	-699.159
2	3	5	0.110	-698.856
3	3	3	0.052	-698.784
4	2	3	0.054	-697.649
5	3	3	0.054	-697.549
6	3	3	0.054	-697.194
7	4	5	0.110	-697.154
8	3	5	0.111	-697.046
9	3	3	0.051	-697.014
10	3	3	0.051	-696.726

Table S8 Results of the grid search for 1 threshold based on the data from January 2005 to December 2017

Number	p_1	p_2	AIC	BIC	th	gamma
1	3	5	-1231.096	-1194.498	0.040	77.154
2	4	5	-1230.487	-1190.839	0.036	77.154
3	2	5	-1230.476	-1196.928	0.037	77.154
4	5	4	-1229.732	-1190.084	0.036	77.154
5	5	2	-1229.515	-1195.967	0.039	77.154
6	1	5	-1229.473	-1198.974	0.024	77.154
7	5	5	-1228.635	-1185.937	0.035	77.154
8	5	3	-1228.633	-1192.035	0.039	77.154
9	5	1	-1228.400	-1197.901	0.023	77.154
10	1	4	-1216.865	-1189.416	0.027	77.154

Table S9 Results of the grid search for the possible LSTAR methods based on the data from January 2005 to December 2017
